# Assessment of flexural strength, diametral tensile strength and microhardness among Cention-N, resin-modified glass ionomer cement, and resin composite: An *in vitro* study

**DOI:** 10.6026/973206300214553

**Published:** 2025-12-15

**Authors:** Jugal Kishore Das, Ajay Kumar Nagpal, Mutiur Rahman, Prashasti Pandey, Somiya Gupta, Seemran Panda

**Affiliations:** 1 Department of Conservative Dentistry and Endodontics, K.D Dental College and Hospital, Mathura, Uttar Pradesh, India

**Keywords:** Flexural strength, diametral tensile strength, tooth-colored restorative materials

## Abstract

The use of mechanically strong restorative materials in posterior teeth is still a clinical issue in modern dentistry. Therefore, it is of interest to compare the flexural strength, diametral tensile strength and surface microhardness of three tooth-colored restorative materials, which are Cention-N (alkasite-based material), Beautifil II LC (giomer) and Ionolux (resin-modified glass ionomer cement). A total of 90 shaped cylindrical samples were produced and put under mechanical testing procedures. Statistically, Cition N has been found to have a much better flexural strength (58.33-1.74 Mpa), microhardness (55.61-8.25 Hv) and still had similar diametral tensile strength as the giomer formulation. Thus, we show that restorative materials composed of alkasite have desirable mechanical characteristics that can be used in loading of the posterior part of the tooth.

## Background:

Dental caries is a consistent worldwide health risk among the people of all age groups and requires an effective restorative intervention that promotes a balance between mechanical and biological compatibility [1]. To restore posterior dentition, there are unique clinical issues related to large occlusal forces of between 200 and 900 Newtons in normal masticatory activity [2]. Therefore, mechanical integrity of restorative materials is an important measure of the long-term clinical success [3]. Conventional dental amalgam has long served as a reference standard of the posterior restorations due to unparalleled activity in terms of durability and good handling qualities [4]. Nevertheless, there has been a dramatic paradigm shift in dental practice in the modern world of patient expectations, environmental issues of mercury content and the practices of minimally invasive dentistry [5] using aesthetic tooth-like materials. This shift has elicited vast studies of alternative material to offer the same mechanical performance but with better aesthetic results [6]. Resin-modified glass ionomer cements (RMGICs) were designed to have the beneficial properties of traditional glass ionomers such as chemical bonding to tooth structure and fluoride release in addition to improved mechanical properties acquired through resin constituents [7]. Regardless of these changes, RMGICs still exhibit weaknesses in flexural strength and wear ability, which limit their use in high stress bearing areas [8]. The use of 2-hydroxyethyl methacrylate (HEMA) has enhanced the handling capabilities, but has not sufficiently resolved underlying mechanical shortcomings [9]. Giomers is an evolutionary development of aesthetic restorative dentistry, which utilizes the technology of surface pre-reacted glass ionomer (S-PRG), to produce hybrid materials that theoretically have the aesthetic properties of resin composite with bioactive properties of glass ionomers [10]. These materials have fluoroboroaluminosilicate glass particles that react with polyalkenoic acid on the surface before being incorporated into a resin matrix [11]. Clinical studies have shown that giomers have medium-term positive results on the posterior restorations [12]. The latest development in dental material science is the introduction of the alkasite-based restorative materials. Cention-N is a commercially available alkasite material, the filler composition of which is based on the inclusion of alkaline components of the glass, which liberate the calcium, fluoride and hydroxide ions [13]. The organic matrix consists of urethane dimethacrylate (UDMA), aromatic aliphatic dimethacrylate and polyethylene glycol-400 dimethacrylate and together, they form a vast network of cross-linked (polymerization) during the process of creating the final product [14]. Manufacturers are purporting to have benefits over conventional glass ionomer cements and uses to bulk-fills [15]. Flexural strength is an important parameter in complex masticatory loaded materials, which mimic bending stresses that occur during the functioning and generates informative predictions about clinical functioning [16]. On the same note, measurements of diametral tensile strength provide information on the material behavior to crack propagation and wear under tensile stress situation that occurs at restoration margins [17]. Surface microhardness measurements are associated with wear resistance and anatomical form maintenance within anatomical structures during functional loading [18]. Although comparative studies evaluating the properties of individual materials of newer generation restorative materials exist, there have been few studies evaluating the mechanical properties of multiple materials simultaneously in the different material categories [19]. This information gap prevents evidence-based choice of material to be used by clinical practitioners who want to use the best possible methods of restoring the posterior teeth. Therefore, it is of interest to assess and compare flexural strength, diametral tensile strength and surface microhardness of alkasite restorative material, a giomer and resin-modified glass ionomer cement under controlled laboratory conditions.

## Materials and Methods:

This research constituted an *in vitro* experimental study designed to evaluate and compare the mechanical properties of three distinct restorative dental materials. The investigation was conducted within the Department of Conservative Dentistry and Endodontics at K.D. Dental College and Hospital, Mathura, following approval from the Institutional Review Board (IRB). All testing procedures were subsequently performed at Lokmani Technical Consultancy, Ghaziabad, Uttar Pradesh, India, ensuring standardized environmental conditions and equipment calibration throughout the experimental phase.

## Sample size calculation and allocation:

The sample size determination was performed a priori using G*Power software (version 3.1.9.2), employing an F-test ANOVA model for fixed effects with omnibus one-way analysis. The calculation was based on a moderate effect size (f = 0.50), significance level (α) set at 0.05, and desired statistical power of 90%. The analysis yielded a total required sample size of 90 specimens, distributed across nine subgroups with an allocation ratio of 1:1:1. This resulted in the recruitment of thirty samples per material group, with each group further subdivided into ten specimens for each of the three mechanical tests (flexural strength, diametral tensile strength, and microhardness), ensuring adequate statistical power (actual power = 0.9227, critical F = 2.0549, noncentrality parameter λ = 22.5000, numerator df = 8, denominator df = 81).

## Materials and specimen preparation:

Three commercially available restorative materials were selected for comparison: Group I consisted of Cention-N (Ivoclar, Switzerland), a compomer material; Group II comprised Beautifill II LC (Shofu, Japan), a nanohybrid composite; and Group III utilized Ionolux (Voco, Germany), a resin-modified glass ionomer cement. Specimen fabrication was performed using standardized circular acrylic molds measuring 4mm in diameter and 6mm in height. The incremental technique was employed for material placement into the molds, with each increment being carefully condensed to minimize void formation. Following filling, the specimens were covered with polyester Mylar strips and sandwiched between glass plates to ensure uniform surface morphology and prevent oxygen inhibition of the polymerization reaction. Photopolymerization was accomplished using a Woodpecker LED curing unit (China) with a standardized exposure time of 30 seconds per increment. After curing, excess flash material was meticulously removed using a sharp GDC instrument kit (India) to achieve precise dimensional conformity.

## Testing procedures:

Three distinct mechanical tests were conducted on the prepared specimens. For flexural strength evaluation, samples were positioned on a two-point support system in the universal testing machine, ensuring precise alignment such that the bar width was oriented perpendicular to the applied force. Testing was performed at a controlled crosshead speed of 1mm/min until catastrophic fracture occurred, with the maximum force recorded in megapascals (MPa). The diametral tensile strength (DTS) assessment involved subjecting the cylindrical specimens to compressive loading along their longitudinal axis at the same crosshead speed of 1mm/min, generating tensile stresses perpendicular to the vertical plane passing through the specimen's center. The resulting tensile strength was similarly quantified in MPa. Microhardness testing was conducted using a Vickers microhardness machine (Lokmani Technical Consultancy) equipped with a diamond indenter shaped as a right pyramid with a square base. All specimens were securely fixed in a specialized holder with the test surface oriented perpendicular to the indenter tip. A load of 50gf was applied for a dwell time of 15 seconds, and the resulting indentation dimensions were used to calculate Vickers hardness numbers.

## Study instruments and equipment:

The experimental armamentarium included the three test materials (Cention-N, Beautifill II LC, Ionolux), a Woodpecker LED curing device, precision circular acrylic molds, glass slabs for specimen stabilization, Mylar strips for oxygen barrier formation, a universal testing machine for strength determinations, and a Vickers microhardness tester. All equipment was calibrated according to manufacturer specifications prior to initiation of the study.

## Ethical considerations and pilot study:

Although an *in vitro* investigation, ethical approval was obtained from the Institutional Review Board of K.D. Dental College and Hospital, Mathura, ensuring adherence to institutional research governance standards. A pilot study was conducted prior to the main experiment to establish feasibility, verify material handling protocols, confirm equipment functionality, and validate the specimen preparation technique. The pilot phase yielded desirable results and confirmed that all required materials were readily available and could be conveniently procured for the full-scale investigation.

## Data collection and statistical analysis:

Data collection was executed systematically following the successful completion of the pilot phase. All experimental procedures were performed in accordance with the standardized protocols established during the pilot study. The collected data were tabulated and subjected to statistical analysis using SPSS software. Intergroup comparisons were performed using one-way analysis of variance (ANOVA) to identify significant differences among the three material groups for each mechanical property. When ANOVA indicated statistically significant differences, post-hoc pairwise comparisons were conducted using Tukey's test to determine specific group-to-group variations. The significance level was established at α = 0.05 for all statistical tests, providing a robust framework for interpreting the mechanical performance differences among the tested restorative materials.

## Results:

Equal distribution of specimens across the three study groups ensured balanced comparison, with each group containing 30 specimens (33.3%). Flexural strength measurements revealed substantial variation among materials. Cention-N demonstrated the highest mean flexural strength (58.33±1.74 MPa, range: 55.46-61.21 MPa), followed by Beautifil II LC (52.54±5.07 MPa, range: 47.46-62.21 MPa). Ionolux exhibited markedly lower flexural strength (5.65±1.50 MPa, range: 4.13-8.88 MPa). One-way ANOVA indicated statistically significant differences among groups (F=807.841, p<0.01). Tukey's post hoc analysis revealed that all pairwise comparisons were statistically significant. The mean difference between Cention-N and Ionolux was 52.68 MPa (p<0.01, 95% CI: 49.12-56.24), between Cention-N and Beautifil II LC was 5.79 MPa (p=0.001, 95% CI: 2.23-9.36) and between Beautifil II LC and Ionolux was 46.89 MPa (p<0.01, 95% CI: 43.32-50.45) (Table 1 - see PDF). Diametral tensile strength measurements demonstrated a different performance pattern. Beautifil II LC exhibited the highest mean value (54.81±3.05 MPa, range: 51.43-60.02 MPa), followed closely by Cention-N (52.48±5.25 MPa, range: 45.68-59.33 MPa). Ionolux showed substantially lower performance (23.34±1.71 MPa, range: 21.10-26.20 MPa). ANOVA testing confirmed significant differences among groups (F=231.563, p<0.01). Post hoc comparisons revealed that while Cention-N and Beautifil II LC did not differ significantly (mean difference: -2.32 MPa, p=0.342, 95% CI: -6.36 to 1.72), both materials demonstrated significantly higher diametral tensile strength compared to Ionolux. The mean difference between Cention-N and Ionolux was 29.15 MPa (p<0.01, 95% CI: 25.10-33.19) and between Beautifil II LC and Ionolux was 31.47 MPa (p<0.01, 95% CI: 27.43-35.51) (Table 2 - see PDF). Surface microhardness testing reveals distinct differences among materials. Cention-N achieved the highest mean microhardness value (55.61±8.25 HV, range: 45.90-71.20 HV), followed by Beautifil II LC (42.62±4.54 HV, range: 36.40-48.30 HV). Ionolux demonstrated the lowest microhardness (15.02±1.29 HV, range: 13.20-17.20 HV) (Table 3 - see PDF). One-way ANOVA demonstrated significant differences among groups (F=142.710, p<0.01). Tukey's post hoc test indicated that all pairwise comparisons were statistically significant. The mean difference between Cention-N and Ionolux was 40.59 HV (p<0.01, 95% CI: 34.51-46.67), between Cention-N and Beautifil II LC was 12.99 HV (p<0.01, 95% CI: 6.91-19.07) and between Beautifil II LC and Ionolux was 27.60 HV (p<0.01, 95% CI: 21.52-33.68).

## Discussion:

The current study offers a detailed comparative analysis of three different types of aesthetic restorative materials, which show large differences in mechanical properties and with some vital clinical implications. The high flexural strength and microhardness of Cention-N coupled with similarity of its diametral tensile strength with giomer material can indicate future benefits in practice in stress-bearing posterior restorations. This very high flexural strength exhibited by Cention-N can be explained by the fact that this particular product is made of alkasite. The organic structure of the material has several dimethacrylate units as its constituents and UDMA is the major constituent with a moderate viscosity and a significant contribution to the mechanical strength due to the large cross-linking during the time of polymerization [2]. This observation is consistent with the past studies that show that alkasite materials have better flexural properties than traditional glass ionomer cements [1]. Inclusion of alkaline fillers i.e. calcium-aluminum-fluorosilicate glass and calcium phosphate compounds, presumably results in higher resistance to bending forces due to better interaction of filler-matrix and higher filler loading [4]. Beautifil II LC had a good flexural strength but much lower compared to Cention-N. This performance is possible due to the use of the S-PRG technology in giomer materials, in which the fluoroboroaluminosilicate glass particles are pre-reacted on the surface with polyalkenoic acid before being introduced into resin matrix [3]. The surface-modified layer obtained provides the glass core with resistance to degradation by moisture and it preserves good mechanical characteristics [5]. Past clinical trials have already proved the acceptability of giomers in use as posterior restorations and wear rates and fracture resistance were similar to traditional resin composites in the medium term assessment periods [6]. Inherent resin-modified glass ionomer cement flaws are manifested in the greatly lower flexural strength of Ionolux. Although the incorporation of photopolymerizable components and others to enhance mechanical properties was done, RMGIC still show brittle nature and low strength as compared to resin-based materials [7]. The quick development of polymer networks might inhibit full acid-base interactions and hinder the best cross-linking of polyalkenoic acid, which causes a weakening of mechanical integrity [8]. Such results can be related to the literature that reports that the flexural strength of RMGICs generally lies between 15-40 Mpa which is much lower than resin composites [9].

The achieved patterns are interesting as diametral tensile strength results showed that Beautifil II LC had slightly higher scores than those of Cition N, although they were not statistically significant. This parameter shows material resistance to crack initiation and propagation under tensile stress, especially at the restoration margins where stress concentrations happen [10]. The similarity in the performances of these two materials implies that they have similar resistance to marginal fracture and debonding when subjected to clinical load conditions. The tensile properties of both materials probably are high since of the good distribution of stress in the material matrix due to the high filler content in the materials [11]. Ionolux exhibited considerably less tensile strength in the form of diametral strengths than was the case with the two composite based materials, proving that it was not suitable in high stress applications. These findings are in line with studies that have found that glass ionomer-based materials usually have a tensile strength of 15-30 Mpa which does not provide the material with the ability to support a load bearing restoration [12]. Measures of surface microhardness indicated that Cention-N gave the highest results, which were far beyond Beautifil II LC and just about out of the sight of Ionolux. Microhardness is strongly associated with wear resistance and wears anatomy preservation during functional loading and tooth brushing abrasion [13]. The better microhardness of Cention-N could be a manifestation of good polymerization efficiency and high integration of polymer fillers, which are a combination of synergistic effects of its various dimethacrylate components and alkaline glass fillers [14]. It has been proved that the materials which have a higher value of the microhardness provided have a better clinical service life and reduced occlusal wear during longer service duration [15]. Beautifil II LC has a moderate microhardness similar to the range of values presented in the literature on nanohybrid composites, which may be found between 35-60 HV, depending on the filler properties and resin content [16]. S-PRG technology, even though offers bioactive properties provided through ion release, could deteriorate surface hardness relative to conventional inorganic fillers in favor of the hydrated surface layer that was present at the stage of the acid-base reaction [17]. The substantially reduced microhardness of Ionolux is an indication of innate softness of glass ionomer-based materials, which include hydrophilic substances and continue to mature through continued maturation of acid-base processes which may go on weeks after the initial setting [18]. The trait makes RMGICs more vulnerable to wear at the surface in the oral cavity, especially in early stages of maturation when moisture contamination can cause serious damage to mechanical properties [19]. Clinically, the results indicate that Cention-N is a good alternative to stress bearing posterior restorations, both Class I and Class II cavities of permanent dentition. It has mechanical property that is similar or higher than those that are usually stated with the conventional resin composites but has potential benefits of being able to be used as a bulk fill and of being less sensitive to technique. Beautifil II LC would still be suitable in use in posterior restorations where both bioactive features and fluoride release is required and where mechanical performance is sufficient most clinically. Low stress applications like Class V restorations, provisional restorations and where the chemical adhesion and release of fluoride properties of the ionolux overrides the mechanical defects should be left to these RMGICs.

## Conclusion:

Cention-N exhibited the best mechanical characteristics such as the greatest flexural strength and microhardness of the materials studied with diametral tensile strength similar to that of giomer formulation. Thus, we show the possible applicability of alkasite-based products as mechanically effective substitutes of stress-bearing posterior restorations in modern dentistry.
